# FABP4 and I-FABP Levels in Pregnant Women Are Associated with Body Mass Index but Not Gestational Diabetes

**DOI:** 10.1155/2022/1089434

**Published:** 2022-05-20

**Authors:** Tamara Vorobjova, Aili Tagoma, Ija Talja, Helis Janson, Anne Kirss, Raivo Uibo

**Affiliations:** ^1^Department of Immunology, Institute of Biomedicine and Translational Medicine, University of Tartu, Estonia; ^2^Women's Clinic, Tartu University Hospital, L. Puusepa 8, Tartu 51014, Estonia

## Abstract

**Objectives:**

Gestational diabetes mellitus (GDM) is glucose intolerance detected initially during pregnancy. GDM poses an increased risk for the development of diabetes later in life. Fatty acid-binding protein 4 (FABP4) is a regulator of lipid metabolism and is associated with obesity, insulin resistance, and type 2 diabetes. Increased level of intestinal fatty acid-binding protein (I-FABP) may indicate impaired intestinal permeability, which may be an important contributor to the pathogenesis of type 1 diabetes and GDM. We aimed to compare FABP4 and I-FABP levels in pregnant women with GDM and in healthy pregnant controls, taking into consideration their prepregnancy body mass index (BMI), past exposures to enteroviruses (EV), and adipokine and cytokine levels, which have been shown to decrease insulin sensitivity. *Material and Methods.* Forty patients with GDM (median age 30.5) and 40 pregnant healthy controls (median age 31.1) were divided on the basis of their prepregnancy BMI into two groups: normal weight (BMI < 25, *n* = 20) and overweight (BMI ≥ 25, *n* = 20). FABP4 and I-FABP were measured from serum samples using commercial ELISA kits.

**Results:**

FABP4 and I-FABP levels did not differ between women with GDM and healthy pregnant controls (*p* > 0.05 for both comparisons). However, both levels were associated with BMI (*p* < 0.001 for both comparisons). Median I-FABP level was the highest in healthy controls with lower BMI (<25) (*p* = 0.0009). FABP4 levels correlated with BMI and C-peptide values in both groups (*p* < 0.001). Anti-EV antibody levels did not correlate with FABP4 or I-FABP levels. FABP4 and adiponectin levels were negatively correlated in controls (*r* = −0.61, *p* = 0.0009), while I-FABP correlated positively with adiponectin (*r* = 0.58, *p* = 0.04) and resistin (*r* = 0.67, *p* = 0.04) levels in the GDM group.

**Conclusion:**

FABP4 and I-FABP levels were not dependent on the diagnosis of GDM, but rather on BMI. The correlation of I-FABP with adiponectin and resistin levels in women with GDM may suggests the importance of lipid metabolism in GDM-associated changes in intestinal permeability.

## 1. Introduction

Gestational diabetes mellitus (GDM) is a glucose intolerance detected initially during pregnancy [1]. The prevalence of GDM in Europe varies from 2 to 6% [1, 2]. In Estonia, GDM was diagnosed in 6% of pregnant women based on glucose tolerance testing (GTT) at gestational weeks 24–28 [3]. GDM poses an increased risk for the subsequent development of both insulin-resistant type 2 diabetes (T2D) and autoimmune type 1 diabetes (T1D) [4].

During normal pregnancy, insulin sensitivity decreases and insulin resistance increases with advancing gestation to ensure fetal energy supply [5, 6]. Development of insulin resistance during pregnancy is dependent on the combined effects of increased maternal adiposity and placental hormones [6]. Immune mediators, including leptin, adiponectin, resistin, TNF-*α*, and IL-6, which are produced by the placenta and maternal white adipose tissue, can decrease insulin sensitivity [7, 8].

Fatty acid-binding protein 4 (FABP4), also known as adipocyte P2, is a member of a superfamily of lipid-binding proteins that was first discovered in adipose tissue and mature adipocytes [9]. FABP4 is a regulator of lipid metabolism and facilitates the transport of fatty acids into cells. FABP4 is associated with obesity, insulin resistance, and T2D [9–11]. FABP4 levels may be significantly increased in GDM patients compared to non-GDM pregnant women [12–14].

Intestinal fatty acid-binding protein (I-FABP) is an intracellular protein specifically expressed in small and large intestinal epithelia [15, 16]. The leakage of I-FABP from the mature villous epithelium into the circulation may indicate intestinal mucosal damage and decreased gut wall integrity [15, 16]. Increased intestinal permeability has been suggested as an important contributor to the pathogenesis of T1D [17, 18]. Elevated levels of zonulin, a biomarker of intestinal permeability, have been observed in women with GDM [19]. In addition, enteroviruses (EV) have been suggested as initiators of the autoimmune pathogenesis of T1D by damaging the small bowel mucosa and pancreatic *β* cells [20–22].

The goal of our present investigation was to compare FABP4 and I-FABP levels in pregnant women with GDM and in healthy pregnant controls, taking into consideration their body mass index (BMI). Furthermore, we aimed to determine whether FABP4 and I-FABP levels are associated with parameters of glucose, insulin, and lipid metabolism as well as with antibodies to EV.

## 2. Materials and Methods

### 2.1. Study Population

Forty patients with GDM (median age 30.5) and 40 healthy pregnant controls (median age 31.1) were selected from a cohort of 473 women (median age 31.0) with singleton pregnancy who were referred consecutively for GTT at gestational weeks 23-31 (median 27.07 for GDM and 25.86 for controls; *p* = 0.01) at Tartu University Hospital Women's Clinic between November 2013 and December 2019. Exclusion criteria were positivity for autoantibodies against protein tyrosine phosphatase, glutamic acid decarboxylase, zinc transporter 8, thyroid peroxidase or tissue transglutaminase, history of cardiovascular disease, use of medications, requirement for *in vitro* fertilization, and delivery of a sick child in the previous pregnancy. Women with GDM and controls were randomly selected and divided on the basis of body mass index (BMI) into two groups: normal weight (BMI < 25 kg/m^2^) and overweight (BMI > 25 kg/m^2^), each comprised of 20 subjects.

Participants fasted for at least 12 hours before GTT. Fasting blood glucose levels were determined in the morning. Participants then drank a solution of 75 g/200 ml glucose, and serum glucose levels were obtained 60 and 120 minutes later. The patients were instructed not to not drink or eat or engage in physical activity during GTT. GDM was diagnosed based on the GTT results according to the International Association of Diabetes in Pregnancy Study Groups Consensus Panel criteria [23]. Serum glucose levels were considered normal if fasting glucose was <5.1 mmol/l, 60-minute glucose level was <10.0 mmol/l, and 120-minute glucose level was <8.5 mmol/l. Postpartum blood samples were drawn after overnight fasting during a visit to the study midwife 6-52 weeks after delivery.

This study was approved by the Ethics Review Committee on Human Research of the University of Tartu (229/M-16, 23.09.2013 and 254/M-16, 21.12.2015) and was conducted in accordance with the Declaration of Helsinki. Written informed consent was obtained from each participant.

### 2.2. Biochemical Analyses

Patient sera were collected and stored at -80°C prior to analyses. Multiple freeze-thaw cycles were avoided. Serum C-peptide levels were measured at the United Laboratories of Tartu University Hospital using a chemiluminescence immunoassay, and results were expressed in nmol/L (reference values 0.37-1.47). Postpartum fasting triglyceride, total serum cholesterol, high-density lipoprotein (HDL) cholesterol, and low-density lipoprotein (LDL) cholesterol levels were determined at the United Laboratories of Tartu University Hospital by using enzymatic colorimetric assays, and values were expressed in mmol/l. The respective reference values were 0.45-2.6 for triglycerides, 2.9-6.9 for total cholesterol, >1.2 for HDL, and 1.2-4.7 for LDL cholesterol.

### 2.3. Evaluation of Serum Fatty Acid-Binding Protein 4 (FABP4) and Intestinal Fatty Acid-Binding Protein (I-FABP)

Serum FABP4 levels were evaluated by using the R&D Systems Quantikine ELISA Kit. Serum samples were diluted at 1 : 20. Results were expressed in pg/ml and calculated according to the manufacturer's instructions (the concentration read from the standard curve has been multiplied by dilution factor 20), I-FABP levels were evaluated by using the Hycult Biotech HK406-02 ELISA Kit. Serum samples were diluted at 1 : 10. Results were expressed in pg/ml and were calculated according to the manufacturer's instructions (the concentration read from the standard curve has been multiplied by dilution factor 10).

### 2.4. Evaluation of Serum IgA and IgG Antibodies to Enterovirus (EV) Peptide Antigen

IgA antibodies to EV peptide antigen were assayed in sera using the ELISA method as described by Viskari et al. [24]. Briefly, microtiter plates (Nunc Immunoplate, Nunc, Glostrup, Denmark) were coated with the synthetic enterovirus peptide (sequence [(NH2) KEVPALTAVETGATC]) derived from an immunodominant region of capsid protein VP1 [25] at 2.5 *μ*g/ml in a carbonate bicarbonate buffer (pH 9.6). The serum samples were diluted at 1 : 100 for detection of IgA antibodies and at 1 : 2000 for detection of IgG antibodies. Results were expressed in enzyme immunoassay units (EIU) which showed the relative antibody reactivity of each sample in relation to the positive and negative reference sera in each assay. A seropositivity cut-off level of 15 EIU was considered significant.

### 2.5. Adipokine and Cytokine Assays

Adiponectin, resistin, leptin, IL-6, and TNF-*α* levels were measured by multiplex analysis using the Milliplex®MAP Magnetic Bead assay according to the manufacturer's instructions (Millipore, Billerica, MA, USA). Measurements were performed by following Milliplex^R^ Map Kit-specific protocols: Human Adipokine Magnetic Bead Panel 1 (96-well Plate Assay Cat # HADK1MAG-61K) for the detection of adiponectin and resistin, Human Adipokine Magnetic Bead Panel 2 (96-well plate assay Cat # HADK2MAG-61 K) for the evaluation of leptin, and Human High Sensitivity T Cell Magnetic Bead Panel (96-well plate assay Cat# HSTCMAG-28SK) for measuring the levels of IL-6 and TNF-*α*. Detection limits varied for each cytokine and ranged from 0.02 ng/ml to 38 pg/ml. According to the manufacturers' specifications, the intra-assay and interassay coefficients of variation were <10% and <20%, respectively, for all assays. Adiponectin, resistin, leptin, IL-6, and TNF-*α* levels were evaluated totally in 38 sera because serum adipokines and cytokines could not be measured from all study subjects, due to the fact that only limited samples were available that had not undergone a previous freeze-thaw cycle.

### 2.6. Statistical Analysis

Results obtained from the different study groups were presented as median with the interquartile (IQR) range (25%-75%). Statistical calculations were performed using the GraphPad Prism 5.0 software, employing the nonparametric Mann–Whitney *U* test (for bivariate comparisons of two continuous variables for nonnormal distribution). For correlation analyses, we used Spearman nonparametric rank correlation analysis and *p* < 0.05 was considered statistically significant. The estimated effect of different parameters on FABP4 and I-FABP levels was analyzed using the general linear model in the jamovi project (2021). *jamovi* (version 1.6) (Computer Software) was retrieved from https://www.jamovi.org and adjusted for GDM diagnosis, age, and prepregnancy BMI.

## 3. Results

### 3.1. Comparison of FABP4 and I-FABP Levels in GDM and Control Groups

**Figure 1:**
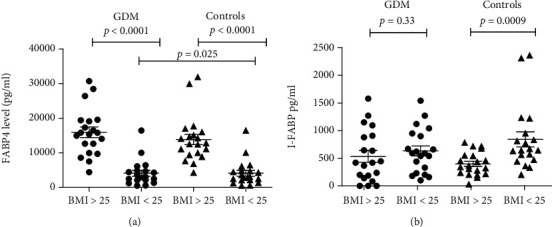
(a) Comparison of FABP4 level in GDM women and in controls based on BMI. In the GDM group BMI > 25 vs. BMI < 25 (15530 (10601-19309) vs. 2930 (2060-4750) pg/ml) (*p* < 0.0001). In controls, BMI > 25 vs. BMI < 25 (12613 (9497-15726) vs. 5730 (3155-9425) pg/ml) (*p* < 0.0001). GDM vs. controls with BMI < 25 (*p* = 0.025). Every dot represents the value of FABP4 in pg/ml for one person. Abbreviations: GDM: gestational diabetes mellitus; FABP4: fatty acid-binding protein 4. (b) Comparison of I-FABP level in GDM women and in controls based on BMI. In the GDM group, BMI > 25 vs. BMI < 25 (42.0 (151.7-900.2) vs. 590 (255.0-937.5) pg/ml) (*p* = 0.33). In controls, BMI > 25 vs. BMI < 25 (353.3 (221.0-547.1) vs. 655.0 (492.5-1153.0) pg/ml) (*p* = 0.0009). The outlier in the control group with I-FABP value of 10050 pg/ml (pregnancy 28 week, 24-year-old healthy female) was excluded from graphic presentation but not from statistical analysis. Every dot represents the value of I-FABP in pg/ml for one person. Abbreviations: GDM: gestational diabetes mellitus; I-FABP: intestinal fatty acid-binding protein.

Table 1 presents the clinical data of the study groups, as well as results of biochemical analyses. Aside from blood glucose levels and gestational week, the two study groups were similar. Although the median serum level of FABP4 was similar in women with GDM (8030.0 pg/ml) and in healthy pregnant controls (9403.0 pg/ml (*p* = 0.67) (Table 1), it was significantly higher in women with BMI > 25 both in the GDM (15530.0 pg/ml vs. 2930.0 pg/ml; *p* < 0.0001) and control groups (12613.0 vs. 5730.0; *p* < 0.0001) compared to women with BMI < 25 (Figure 1(a)). At the same time, median FABP4 level was significantly lower in GDM women with BMI < 25 when compared to the control women with BMI < 25 (2930.0 pg/ml vs. 5730.0 pg/ml; *p* = 0.025 (Figure 1(a)).

I-FABP levels in the GDM (540.0 pg/ml) and control groups (545.0 pg/ml) were similar (*p* = 0.60) (Table 1). However, in the control group, I-FABP levels were significantly higher in women with BMI < 25 compared to women with BMI > 25 (655.0 pg/ml vs. 353.3 pg/ml; *p* = 0.0009) (Figure 1(b)).

There was no significant correlation between the gestational week when the sera were obtained for analysis and the level of FABP4 neither in GDM group (*r* = −0.08, *p* = 0.60), nor in control group (*r* = 0.12, *p* = 0.43). Also the level of I-FABP did not correlate with gestational week (*r* = −0.06, *p* = 0.70 for GDM and *r* = 0.17, *p* = 0.27 for controls). Also, the general linear model did not show any association between the level of FABP4 or I-FABP with gestational week (*B* = 0.13, *p* = 0.12 for FABP4 and *B* = 0.14, *p* = 0.23 for I-FABP).

We can emphasize that FABP4 and I-FABP levels were similar in GDM and healthy pregnant controls. However, FABP4 levels were significantly higher in women with BMI > 25 in both the GDM and healthy pregnant control groups.

### 3.2. Correlations of FABP4 and I-FABP with Other Covariates

Correlations of FABP4 and I-FABP levels with other measured parameters are presented in Table 2. There was no significant correlation between FABP4 and I-FABP levels in either the GDM group (*r* = 0.004, *p* = 0.97) or in the control group (*r* = −9.26, *p* = 0.10). There was a clear significant positive correlation between FABP4 level and BMI in the GDM group (*r* = 0.68, *p* < 0.0001) as well as in the control group (*r* = 0.73, *p* < 0.0001). On the other hand, I-FABP levels correlated negatively with BMI, but only in the control group (*r* = −0.36, *p* = 0.01) (Table 2). FABP4 and I-FABP levels did not correlate with glucose levels measured during GTT, except for I-FABP level which revealed a negative correlation with GTT 120- minute glucose levels in controls (*r* = −0.33, *p* = 0.03; Table 2).

The median weight gains in the total study group (*n* = 80) was 15.0 (11.0-19.88) kg; in the GDM group (*n* = 40), it was 12.75 (8.87-16.23) kg; and in the control group (*n* = 40), it was 16.0 (12.0-20.0) kg. The weight gain does not differ significantly in the GDM and control groups (*p* = 0.11). The weight gain does not correlate with the level of FABP4 neither in GDM (*n* = 40; *r* = −0.08, *p* = 0.58) nor in the control group (*n* = 40; *r* = −0.12, *p* = 0.43). The level of I-FABP correlated with weight gain in the total group studied (*n* = 80; *r* = 0.29, *p* = 0.008). In the GDM group, the weight gain did not correlate significantly with the level of I-FABP (*n* = 40; *r* = 0.30, *p* = 0.056); also, in the control group, this correlation was not significant (*n* = 40; *r* = 0.30, *p* = 0.05).

**Figure 2:**
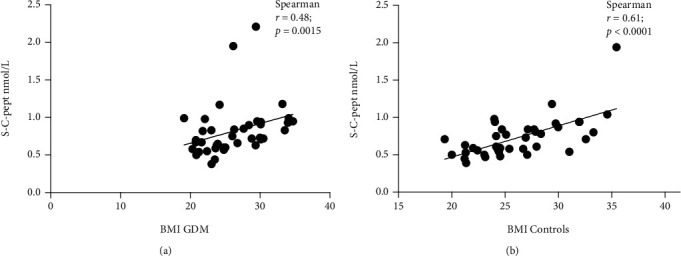
(a) Correlation between C-peptide level and BMI in GDM women. (b) Correlation between C-peptide level and BMI in controls.

FABP4 levels correlated significantly with C-peptide level in the GDM (*r* = 0.70, *p* < 0.0001) and control groups (*r* = 0.50, *p* = 0.001) and also in postpartum samples (Table 2). On the other hand, I-FABP showed no correlation with C-peptide level (Table 2). We controlled whether the C-peptide value correlated with BMI in our study participants and found a significant correlation in both groups (in GDM, *r* = 0.48, *p* = 0.0015; in controls, *r* = 0.61, *p* < 0.0001; Figures 2(a) and 2(b)).

**Figure 3:**
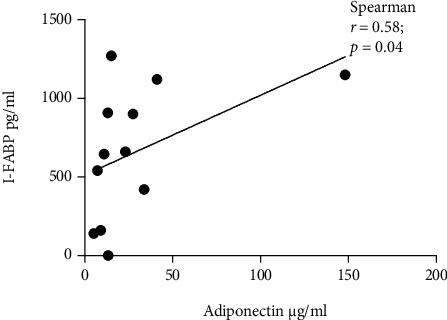
Correlation between I-FABP and adiponectin level in the GDM group. The data for adiponectin level were available in 12 GDM samples.

**Figure 4:**
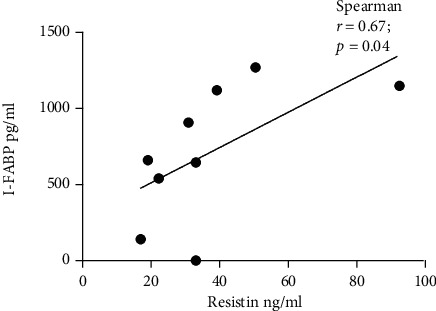
Correlation between I-FABP and resistin level in the GDM group. The data for resistin level were available in 9 GDM samples.

We observed a significant negative correlation between adiponectin and FABP4 levels in controls (*r* = −0.61, *p* = 0.0009) (Table 2). On the other hand, I-FABP correlated positively with adiponectin (*r* = 0.58, *p* = 0.04) and resistin (*r* = 0.67, *p* = 0.04) levels, but only in the GDM group (Figures 3 and 4). The level of IgA or IgG antibodies to EV did not correlate with FABP4 or I-FABP levels in any of the study groups (Table 2).

FABP4 correlated significantly with triglyceride levels in control women (*r* = 0.43, *p* = 0.005; Table 3), but not in women with GDM (*r* = 0.05, *p* = 0.72; Table 3). HDL cholesterol was negatively correlated with FABP4, but only when all the study participants were considered as one group (*n* = 80; *r* = −0.29, *p* = 0.007; Table 3). We also found a negative correlation between HDL cholesterol values and BMI (*r* = −0.23, *p* = 0.03) (Table 3).

Taken together, the most important results of this correlation study are that FABP4 levels correlated significantly with BMI and C-peptide levels, both in the GDM and control groups. I-FABP level was significantly correlated with adiponectin and resistin levels in the GDM group.

### 3.3. Linear Regression Models

We used general linear models to evaluate interaction effects between the covariates and the levels of FABP4 and I-FABP, adjusted for diagnosis. We found positive associations of FABP4 level with BMI (*B* = 51.75, *p* < 0.001), age (*B* = 15.76, *p* = 0.03), and TNF-*α* level (*B* = 24.42, *p* = 0.011) (ad*R*^2^ = 0.58), irrespective of GDM diagnosis (*p* = 0.63). I-FABP levels showed positive associations with IL-6 (*B* = 32.85, *p* = 0.002) and TNF-*α* (*B* = 13.2, *p* = 0.03), but an inverse association with age at the beginning of the study (*B* = −10.03, *p* = 0.04) (ad*R*^2^ = 0.39), irrespective of GDM diagnosis (*p* = 0.26).

The most important finding of the general linear models was that irrespective of GDM diagnosis, positive associations of FABP4 level were found with BMI, age, and TNF-*α* level, whereas I-FABP level was positively associated with IL-6 and TNF-*α* levels.

## 4. Discussion

The essential finding of our study was that neither FABP4 nor I-FABP levels differed significantly between the GDM and control groups. However, FABP4 level correlated significantly with BMI in both groups and was significantly higher in women with BMI > 25. The serum level of I-FABP showed a significant negative correlation with BMI in controls.

Significant elevations of FABP4 levels have been reported in patients with GDM compared with healthy pregnant controls [5, 14, 26–29]. This increase is primarily due to FABP production by adipocytes and also by the placenta and is related to enhanced lipolysis and exacerbation of insulin resistance during pregnancy [15, 30]. In our study, FABP4 level was independent of GDM diagnosis and was associated with higher BMI in both the GDM and control groups. This finding is consonant with other studies that have associated FABP4 with obesity [26–28], as well as by the linkage of higher BMI values and GDM [31, 32]. Interestingly, women with GDM and BMI < 25 showed the lowest FABP4 levels, even when compared to healthy controls. Although higher BMI is one of the major risk factors for GDM [31, 32], our result suggests that the etiology of GDM in women with normal BMI might be related to other factors.

Apart from BMI, FABP4 has been associated with other markers and risk factors for adiposity, for example, triglycerides, leptin, and cholesterol levels [26, 33–35]. FABP4 and fatty acids promote and regulate insulin secretion during obesity [36, 37]. Moreover, FABP4 has been correlated with C-peptide [37], which denotes endogenous insulin production [38]. Our results confirmed the association of FABP4 and C-peptide levels. In addition, we found a positive correlation between FABP4 and triglyceride levels in healthy control women. On the other hand, HDL cholesterol level was negatively correlated with FABP4. Lower levels of HDL cholesterol could be related to the metabolic syndrome [39] or to the influence of BMI, since we also detected a negative correlation between HDL cholesterol and BMI.

Proinflammatory cytokines and adipokines modulate pathways for insulin signaling, lipid metabolism, inflammatory response, and pathogenesis of GDM [11, 27, 40–43]. For example, IL-6 directly inhibits insulin sensitivity and upregulates the release of hormones that contribute to insulin resistance via induction by insulin receptor's signal transduction in hepatocytes [44, 45]. Although a longitudinal study by Francis et al. [43] found significant associations of elevated FABP4, leptin, and IL-6 levels with increased GDM risk, we observed no correlations between FABP4 and IL-6, leptin, or resistin levels. However, using linear regression models, we found a positive association of FABP4 with TNF-*α* levels, irrespective of GDM diagnosis. The potential role of TNF-*α* in the development of insulin resistance during pregnancy was shown by Kinalski et al. [46], who found significantly higher TNF-*α* levels in patients with GDM and proposed early pregnancy BMI as the most predictive indicator of TNF-*α* concentration.

We detected a negative correlation between FABP4 and adiponectin levels in the control group. Adiponectin is secreted by both adipocytes and the placenta [47, 48]. It stimulates glucose utilization and can enhance insulin sensitivity. Due to crosstalk between the placenta, maternal adipose tissue, and *β* cells; adipokines, including adiponectin, can also influence *β* cell function [8, 47–49]. Guelfi et al. [50] reported that maternal adiponectin levels decrease with advancing pregnancy. In light of the inverse association between adiponectin and BMI [31, 51], our finding of a negative correlation between FABP4 and adiponectin was expected and adds further proof that higher FABP4 levels are due to higher BMI levels in these women.

Increased I-FABP has been proposed as a biomarker for intestinal barrier dysfunction in patients with varying durations of hyperglycemia. A positive association of serum I-FABP level with duration of hyperglycemia and a negative association with islet *β* cell function has been reported in patients with different courses of diabetes [52, 53]. In our study, I-FABP was inversely correlated with the 120-minute glycose level in controls.

The significant correlation of I-FABP with adiponectin and resistin levels in women with GDM in our study could indicate altered intestinal permeability, corresponding to the findings of Hogan et al. [54], who described the role of resitin-like molecule *β* in the maintenance of colonic barrier function and intestinal innate immune response. Resistin, originally described by Steppan et al. [55] as a unique signaling molecule secreted by adipocytes contributes to insulin resistance and has been suggested as a link between obesity and diabetes.

Furthermore, our study demonstrated an increasing effect of proinflammatory IL-6 and TNF-*α* levels on I-FABP levels. Similarly, a link between increased markers of proinflammatory cytokine response and an elevated marker of intestinal permeability (zonulin) in patients with T2D has been proposed [56]. Increased intestinal permeability may lead to translocation of lipopolysaccharides from the intestinal lumen, which activate proinflammatory cytokines. The role of a high-fat diet in the alteration of the gut microbiota and in the induction of gut inflammation was also proposed in a report by de La Serre et al. [57].

Because intestinal EV infections increase intestinal permeability [17, 20], we hypothesized a correlation between antibodies against a common EV peptide (indicating past EV infection) and intestinal permeability. However, neither FABP4 nor I-FABP levels correlated with IgG or IgA antibodies to EVs. Thus, our results do not support our preliminary hypothesis regarding a possible link between increased intestinal permeability and previous EV infection with GDM development.

## 5. Conclusions

Serum levels of both FABP4 and I-FABP were unrelated to a diagnosis of GDM but depended rather on BMI. FABP4 level was positively associated with BMI and TNF-*α* level. I-FABP, on the other hand, showed an inverse association with BMI in controls and was positively associated with IL-6 and TNF-*α* levels. The significant correlation of I-FABP level with adiponectin and resistin levels in women with GDM may indicate the importance of lipid metabolism in GDM-associated changes in intestinal permeability. No association was revealed between FABP4 or I-FABP levels and antibodies to EV.

## Figures and Tables

**Table 1:** Clinical parameters of patients with GDM and healthy pregnant control women.

Pregnancy parameter	GDM, *n* = 40		Controls, *n* = 40		Mann–Whitney
	Median	(25%-75%)	Median	(25%-75%)	*p* value
Age (years)^a^	30.5	(28.0-35.0)	31.1	(27.25-31.0)	0.87
Gestational week^a^	27.07	(25.57-28.21)	25.86	(24.75-26.96)	0.01
BMI (kg/m^2^)	25.53	(22.56-29.89)	24.77	(22.96-28.97)	0.79
FABP4 level (pg/ml)	8030	(2885-15784)	9403	(5515-13147)	0.67
I-FABP level (pg/ml)	540.0	(199.4-905.6)	545.0	(322.5-756.55)	0.6
Fasting glucose (mmol/l)	5.0	(4.7-5.27)	4.5	(4.22-4.7)	<0.0001
60′ glucose (mmol/l)	10.15	(7.75-10.78)	6.85	(5.4-7.75)	<0.0001
120′ glucose (mmol/l)	7.4	(5.82-8.7)	5.85	(5.1-6.47)	0.0001
C-peptide (nmol/l)^b^	0.72	(0.60-0.93)	0.71	(0.54-0.84)	0.13
IL-6 (pg/ml)^c^	2.55	(1.14-3.69)	1.41	(0.89-1.90)	0.1
TNF-*α* (pg/ml)^d^	2.97	(2.21-4.91)	3.71	(2.64-6.92)	0.31
Adiponectin (*μ*g/ml)^d^	14.28	(9.61-32.25)	22.54	(15.01-31.87)	0.32
Leptin (ng/ml)^d^	28.97	(23.39-40.65)	25.44	(18.53-39.88)	0.49
Resistin (ng/ml)^e^	33.08	(20.68-44.81)	31.99	(22.53-38.06)	0.85
EV IgA (EIU)	12.35	(1.92-27.80)	9.95	(4.25-28.33)	0.91
EV IgG (EIU)	7.55	(2.1-19.28)	11.7	(0.1-23.80)	0.48

*Postpartum parameters*					
Fasting glucose (mmol/l)	4.95	(4.72-5.1)	4.70	(4.3-5.0)	0.009
C-peptide (nmol/l)	0.59	(0.50-0.75)	0.61	(0.47-0.74)	0.82
Triglycerides (mmol/l)	0.89	(0.61-1.13)	0.77	(0.68-1.20)	0.83
Cholesterol (mmol/l)^f^	5.4	(4.5-6.1)	5.05	(4.32-5.87)	0.38
HDL cholesterol (mmol/l)	1.65	(1.43-2.07)	1.68	(1.39-1.95)	0.5
LDL cholesterol (mmol/l)	3.58	(2.72-4.39)	3.25	(2.60-3.68)	0.16

^a^Continuous data is presented as median (25%-75% percentile) value. ^b^Data available for 40 GDM and 39 control women. ^c^Data available for 9 GDM and 26 control women. ^d^Data available for 12 GDM and 26 control women. ^e^Data available for 9 GDM and 29 control women. ^f^Data available for 39 GDM and 40 control women.

**Table 2:** Spearman correlations of FABP4 and I-FABP levels with other parameters.

Parameter		FABP4			I-FABP		
		GDM, *n* = 40	Controls, *n* = 40	Whole group, *n* = 80	GDM, *n* = 40	Controls, *n* = 40	Whole group, *n* = 80
BMI (kg/m^2^)	*r*	0.68	0.70	0.69	-0.08	-0.35	-0.21
	*p*	<0.0001	<0.0001	<0.0001	0.60	0.02	0.06
Fasting glucose (mmol/l)	*r*	0.06	0.26	0.11	0.12	0.07	0.04
	*p*	0.7	0.09	0.3	0.44	0.66	0.7
Postpartum fasting glucose (mmol/l)	*r*	0.14	0.03	0.10	-0.05	0.06	-0.01
	*p*	0.37	0.81	0.37	0.73	0.69	0.88
60′ glucose (mmol/l)	*r*	-0.13	0.24	-0.02	0.05	-0.02	-0.01
	*p*	0.38	0.12	0.79	0.75	0.85	0.86
120′ glucose (mmol/l)	*r*	-0.14	0.23	-0.03	0.07	-0.33	-0.1
	*p*	0.37	0.13	0.74	0.65	0.03	0.33
C-peptide (nmol/l)^a^	*r*	0.7	0.5	0.58	-0.005	-0.13	-0.08
	*p*	<0.0001	0.001	<0.0001	0.97	0.4	0.44
Postpartum C-peptide (nmol/l)	*r*	0.37	0.45	0.40	-0.16	-0.06	-0.14
	*p*	0.01	0.003	0.0002	0.3	0.69	0.20
IL-6 (pg/ml)^b^	*r*	-0.13	0.26	0.2	-0.39	-0.11	-11
	*p*	0.68	0.18	0.22	0.2	0.56	0.48
TNF-*α* (pg/ml)^b^	*r*	0.08	0.23	0.21	0.06	0.06	0.09
	*p*	0.79	0.24	0.18	0.82	0.77	0.58
Adiponectin (*μ*g/ml)^b^	*r*	0.19	-0.61	-0.35	0.58	0.17	0.28
	*p*	0.54	0.0009	0.02	0.04	0.39	0.08
Leptin (ng/ml)^b^	*r*	0.26	0.08	0.19	0.32	0.08	0.11
	*p*	0.4	0.66	0.23	0.29	0.69	0.49
Resistin (ng/ml)^c^	*r*	-0.12	-0.02	-0.01	0.67	-0.02	0.17
	*p*	0.74	0.91	0.9	0.04	0.91	0.29
EV IgA (EIU)	*r*	0.06	0.08	0.03	-0.006	0.13	0.05
	*p*	0.68	0.62	0.77	0.96	0.39	0.61
EV IgG (EIU)	*r*	0.05	0.17	0.06	0.24	-0.16	0.013
	*p*	0.75	0.28	0.57	0.13	0.3	0.9

^a^Data available for 40 GDM and 39 control women. ^b^Data available for 12 GDM and 26 control women. ^c^Data available for 9 GDM and 29 control women.

**Table 3:** Spearman correlations between FABP4 and lipid metabolism parameters.

Parameter		FABP4			BMI
		GDM, *n* = 40	Controls, *n* = 40	Whole group, *n* = 80	Whole group, *n* = 80
Triglycerides (mmol/l)	*r*	0.05	0.43	0.24	0.21
	*p*	0.72	0.005	0.02	0.055
Cholesterol (mmol/l)^a^	*r*	-0.18	-0.01	-0.09	-0.05
	*p*	0.25	0.91	0.43	0.6
HDL cholesterol (mmol/l)	*r*	-0.27	-0.24	-0.29	-0.23
	*p*	0.08	0.12	0.007	0.03
LDL cholesterol (mmol/l)	*r*	-0.1	0.04	-0.03	-0.2
	*p*	0.52	0.79	0.78	0.82

^a^Data available for 39 GDM and 40 control women.

## Data Availability

The data used to support the findings of this study are available from the corresponding authors upon request.
